# Multi-institutional evaluation of isocentricity on a ring-gantry linear accelerator platform with double-stacked MLCs^☆^

**DOI:** 10.1016/j.phro.2026.101008

**Published:** 2026-06-02

**Authors:** Xiaodong Zhao, Phillip D. Wall, Justin Mikell, Matthew C. Schmidt, Eric Laugeman, Cory S. Knill, Dennis N. Stanley, Richard A. Popple, Thomas R. Mazur

**Affiliations:** a Department of Radiation Oncology, Washington University in St. Louis, St. Louis, MO, USA; b Department of Radiation Oncology, University of Michigan, Ann Arbor, MI, USA; c Department of Radiation Oncology, University of Alabama at Birmingham, Birmingham, AL, USA

**Keywords:** Isocentricity, Winston–Lutz, MPC, Ring-gantry linac, Dual-bank double-stacked and staggered MLC

## Abstract

**Background and purpose:**

This study aimed to characterize the isocentricity of ring-gantry linacs and evaluate variability across institutions and software. Isocentricity is assessed using vendor-provided and user-defined Winston–Lutz (WL) tests.

**Materials and methods:**

WL was performed at two institutions on a ring-gantry linac using a custom phantom over 1.5 years. Distal and proximal MLCs were tested, with measurements obtained before and after applying 3D shifts. WL analysis was performed using in-house, commercial (C1–C3), and open-source software. Assessed parameters included the 2D ball bearing (BB)–radiation offset, the 3D offset minimizing 2D errors, and 3D isocenter size. 3D isocenter size was additionally computed using gantry-only fields to remove collimator walkout contributions.

**Results:**

Results across institutions showed sub-millimeter agreement among software except for C1 (max 0.99 mm difference). Machine-dependent variations were observed (p < 0.03). Proximal and distal banks demonstrated small but statistically significant differences (p < 0.03) in 2D/3D offsets and isocenter size, with greater walkout in the proximal bank and poorer axis alignment in the distal bank. Collimator-walkout-corrected 3D isocenter size was not statistically different between proximal and distal bank or between pre- and post-3D-offset shift.

**Conclusion:**

Isocentricity measurements depended on software, MLC bank, pre-/post-offset correction, and institution. For the tested machines, which showed a mean imaging-to-radiation isocenter coincidence of 0.8 mm and a radiation-isocenter size of 0.7 mm, a minimum targeting margin of 1.5 mm would be appropriate to account for geometric uncertainty. With the results from this study, clinicians can choose their own margins based on their machine characteristics.

## Highlights

• Sub-millimeter differences exist in Winston-Lutz results across software and institutions.
• Low contrast image results require careful scrutiny when using certain commercial software.
• Institutions should independently characterize proximal and distal MLC banks separately.
• Collimator-corrected 3D isocenter size is independent of phantom setup or bank.

## Graphical abstract

**Figure:**
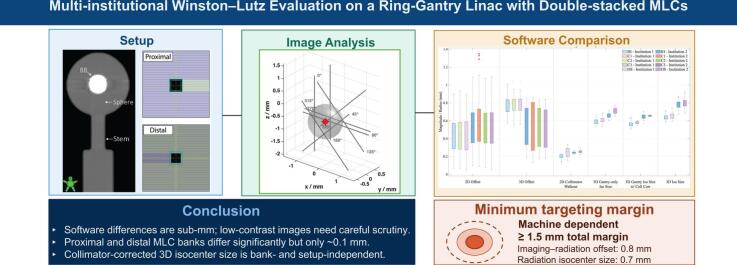

## 1 Introduction

In comparison with standard C-arm linear accelerators (linacs), ring-gantry systems such as the Halcyon (Varian Medical Systems a Siemens Healthineers Company, Inc., Palo Alto, CA) offer more efficient treatment delivery due to increased gantry rotation and multi-leaf collimator (MLC) speeds [1]. These platforms are jaw-less and treat with a 6FFF MV beam at an 800 MU/min maximum dose rate. In lieu of jaws, the treatment field is defined by two stacked MLC banks offset by half the width of an individual leaf (1 cm wide at isocenter). The MLCs consist of 29 leaf pairs in the upper (proximal to radiation source) bank that is closer to the target and 28 leaf pairs in the lower (distal to radiation source) bank. The banks rotate together on a common collimator axis with maximum leaf speed of 5 cm/sec at the isocenter plane. These ring-gantry systems have been used for stereotactic body radiotherapy (SBRT) [2] and stereotactic radiosurgery (SRS), and studies have evaluated single-institution data relating to their isocentricity [3], [4], [5].

The Halcyon platform presents unique challenges for monitoring and maintaining isocentricity compared with conventional C-arm linacs. Unlike C-arm systems, where beam steering can be electronically adjusted, magnetron-based ring-gantry accelerators do not permit RF beam steering. Additionally, beam alignment corrections require mechanical adjustment of the stand assembly, complicating precise beam steering and focal spot alignment. In addition, because the MV and kV imaging systems are mounted to the ring gantry opposite and orthogonal to the source, translating well-established C-arm calibrations such as IsoCal requires additional considerations. The absence of a light field further limits the applicability of traditional mechanical axis checks.

Despite these system-specific challenges, the Winston–Lutz test (WL) [6] remains an effective and comprehensive means for testing both radiation isocenter and mechanical degrees of freedom on ring-gantry linacs. With appropriate plan design, WL can isolate system-level and axis-specific behavior. While a powerful technique, numerous analysis software packages exist with varying underlying computation techniques that can complicate inter-comparison of results both across institutions and software vendors. For example, Winkler et al. [7] introduced a histogram-based center-of-mass method for radiation field centroids and a convolution-based technique for BB localization. Du et al. [8] later demonstrated that Hough transform–based detection is more robust under low signal-to-noise conditions and less affected by image artifacts. Similarly, even definitions of 3D isocenter size vary: some algorithms compute 3D target position from 2D projections of BB-to-field offsets [9], while others back-project central-axis (CAX) rays to determine the minimal enclosing sphere [10]. As a result, a WL measurement can yield different numerical results depending on the analysis method, underscoring the need for greater clarity and standardization when evaluating isocentricity, particularly on newer ring-gantry systems with their unique challenges.

This study characterizes the isocentricity performance of a ring-gantry platform using WL measurements acquired at two institutions with the same phantom customized for this platform. Analyses were performed using multiple software solutions, including in-house, commercial, and open-source tools, to quantify algorithm-dependent variation in reported metrics and to assess how these differences may influence the interpretation of isocentricity results.

## 2 Materials and methods

### 2.1 Phantom setup

**Fig. 1:**
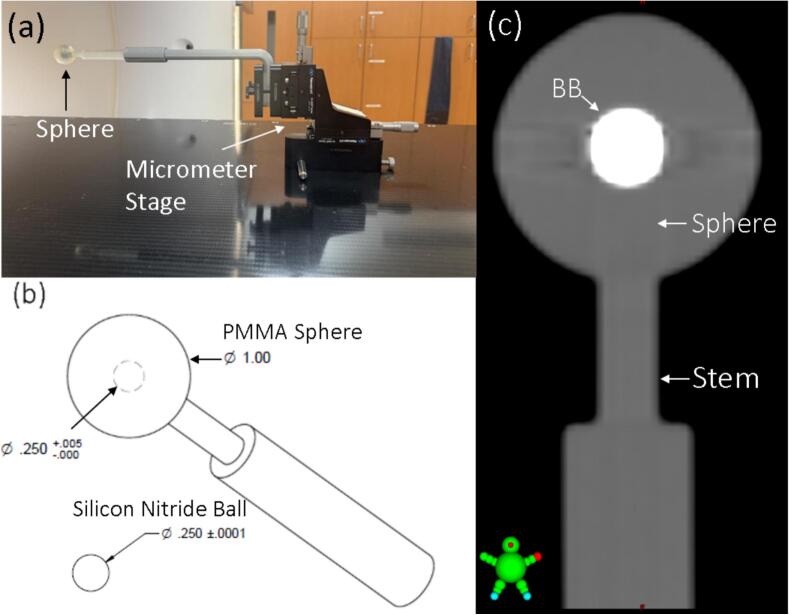
(a) 3D-printed WL phantom mounted on the micrometer; (b) CAD rendering of phantom design specifications, with displayed distances in inches; (c) CT verification of phantom.

WL measurements were performed on separate Varian Ethos platforms at two institutions. As shown in Fig. 1, all measurements were performed with a customized phantom designed specifically for this study and fabricated in-house. We elected not to use a standard WL phantom, as the customized design reduces CBCT imaging (the only available imaging on the Ethos system) artifacts by employing a lower-density silicon nitride BB rather than a conventional high-density tungsten BB. While CBCT-based localization is not strictly required for WL machine isocentricity, it was used in this study to establish the imaging isocenter as the reference frame, allowing direct quantification of the imaging-to-radiation isocenter agreement, which is most relevant for clinical workflows. In addition, the design enables precise delineation of couch-movement uncertainty through sub-millimeter adjustments using a manual micrometer, rather than relying on couch shifts. Couch position was adjusted using a Newport M-460P-XYZ three-axis linear translation stage (Newport Corporation, Irvine, CA). The phantom consists of a 25.40 mm diameter resin sphere with a spherical bore to its centroid and a stem that fits into this bore. A 6.35 mm diameter silicon nitride BB is pressed into the bore of the sphere, and the stem – with a concave face that matches the BB diameter – secures the BB in place within the sphere. The channel is filled with adhesive to prevent any air gaps within the ‘lollipop’ created by the combination of sphere, BB, and stem. The sphere and stem were 3D-printed with photopolymer clear resin on a Formlabs Form 3 stereolithography (SLA) printer (Formlabs, Somerville, MA). The entire phantom was mounted to a three-axis positioning stage, providing sub-millimeter positioning resolution to supplement the couch axes with finer positioning capabilities for the phantom (in the interest of isolating isocentricity characteristics beyond the couch and kV-MV non-coincidence).

The same phantom used across institutions was imaged with a high-resolution CT protocol (0.7 × 0.7 × 0.6 mm^3^) to verify its geometric properties – including its dimensions and coincidence between resin spheres and BB – and to support WL plan design. As the Ethos platform lacks planar radiographic imaging, the BB was localized to the isocenter by CBCT guidance with an axial resolution of 0.55 mm and slice thickness of 2 mm.

### 2.2 Image acquisition

The proximal and distal banks were tested individually by fully retracting the non-tested bank. Distal and proximal MLCs were configured as 4 × 4 cm^2^ and 5 × 5 cm^2^ apertures respectively, such that each bank aperture was centered on the target, accounting for the 5 mm longitudinal leaf offset between banks. For each bank, images were acquired via the electronic portal imaging device (EPID) at eight gantry angles (0°, 45°, 90°, 135°, 180°, 225°, 270°, 315°) with the collimator fixed at 0°, and at seven collimator angles (0°, 30°, 60°, 90°, 270°, 300°, 330°) with the gantry fixed at 0°, resulting in 30 images per session (15 per bank). Multiple applications were used to analyze images including an in-house (IH) software developed in Matlab (MathWorks, Natick, MA), three commercially available solutions (Total QA [C1], Image Owl Inc, Greenwich, NY; DoseLab [C2], Mobius Medical Systems, Houston, TX; and Machine Performance Check [C3], Varian Medical Systems a Siemens Healthineers Company), and an open-source application (OS-Pylinac [11], https://github.com/jrkerns/pylinac). Institution 1′s data excluded C1 due to software access. Institution 1 collected data from February 2023 to June 2024. Institution 2 collected data from May 2023 to October 2023. Isocentricity parameters listed in Table 1 were compared between institutions and software.

**Table 1:** Parameters included in the study. ‘Both’ (green) indicates availability in both institutions; ‘1’ or ‘2’ (pink) indicates availability only in Institution 1 or Institution 2; blank (grey) indicates no data in this category. Detailed calculation procedures and schematic figures for each metric are tabulated in Table S1 in the supplemental material.

### 2.3 3D shifts and statistical analysis

Dual-bank WL tests were acquired pre- and post-correction of 3D offset. The phantom was first aligned with CBCT and WL images were acquired to determine the 3D offset between kV and MV isocenters. This offset was then corrected via micrometer, and a second WL acquisition was performed, with shifts applied independently to the proximal and distal banks. Paired t-tests were performed for comparisons between applications, institutions, banks, and pre- vs post-offset correction. A significance threshold of p < 0.05 was used. Warning and failure limits were defined as 1.96σ and 3σ from the mean for each machine, analysis method, and category.

### 2.4 Collimator walkout dependence on gantry angle

Additionally, we assumed 2D collimator walkout remains constant across different gantry angles. To test this, a series of 56 images were acquired by capturing seven collimator angles at each of eight gantry angles. From these images, the collimator walkout was evaluated at each gantry angle. For each gantry angle, the smallest enclosing circle of the walkout was defined, and the center of this circle was recorded. The collection of gantry-specific collimator centers across all gantry angles was then used to construct a second enclosing circle. Ideally, this circle would collapse to a single point if collimator walkout centers were identical at all gantry angles. Finally, to assess statistical significance, a Mann–Whitney *U* test was performed to compare the walkout circles and radii at gantry 0° versus those at all other gantry angles. P-values less than 0.05 were considered significant.

## 3 Results

### 3.1 Comparisons between software, MLC banks, institutions, and 3D shifts

**Fig. 2:**
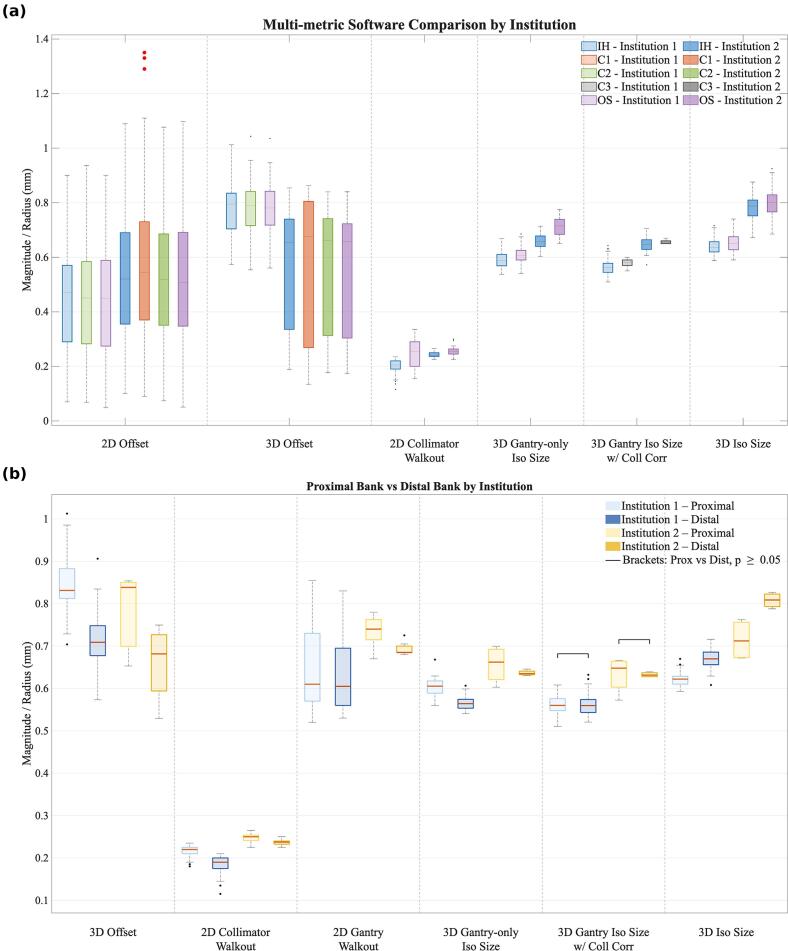

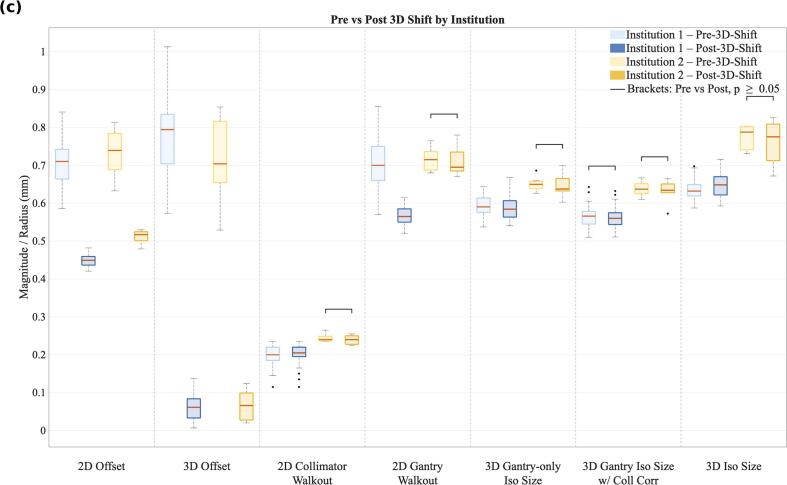
Software, bank, institution, and 3D-shift comparisons. (a) Comparison of metrics across software platforms, stratified by institution. (b) Comparison between proximal vs. distal MLC banks and Institution 1 vs. Institution 2. (c) Comparison of pre- vs. post-3D-shift dataset. Non–statistically significant comparisons are annotated in brackets in (b) and (c); all remaining comparisons were statistically significant (p < 0.05). *IH = in-house Matlab script, C1 = TotalQA, C2 = DoseLab, C3 = MPC, OS = Pylinac.

Fig. 2 summarizes the comparisons across (a) software platforms and institutions for pooled bank data, (b) banks and institutions for pooled software, and (c) pre- vs post-shift and institutions for pooled software and bank data.

In Fig. 2a, differences between software applications were sub-millimeter and not statistically significant. C1 did show clear outliers (differences up to 0.99 mm shown in the supplemental Material) due to incorrect BB-centroid detection resulting from the low image contrast of the silicon nitride BB compared to the standard steel or tungsten BB, so C1 was therefore excluded from subsequent analyses.

Fig. 2b shows statistically significant differences between institutions in all metrics (p < 0.03) and between proximal and distal banks in all metrics (p < 0.03) except 3D gantry-only isocenter size with collimator-walkout correction (p > 0.1). The proximal leaves consistently produced larger offsets or walkout radii than the distal leaves for all metrics except the 3D isocenter size, which increased by less than 0.1 mm.

Fig. 2c demonstrates that applying the 3D shift reduced almost all metrics as expected. For Institution 1, all metrics showed statistically significant pre- vs. post-shift differences (p < 0.05) except the 3D gantry isocenter size with collimator-walkout correction (p > 0.3). For Institution 2, only the 2D and 3D offset metrics reached statistical significance (p < 0.001), while the remaining metrics did not (p > 0.46), likely reflecting limited statistical power due to the smaller sample size.

### 3.2 Ring-gantry isocentricity characteristics

**Fig. 3:**
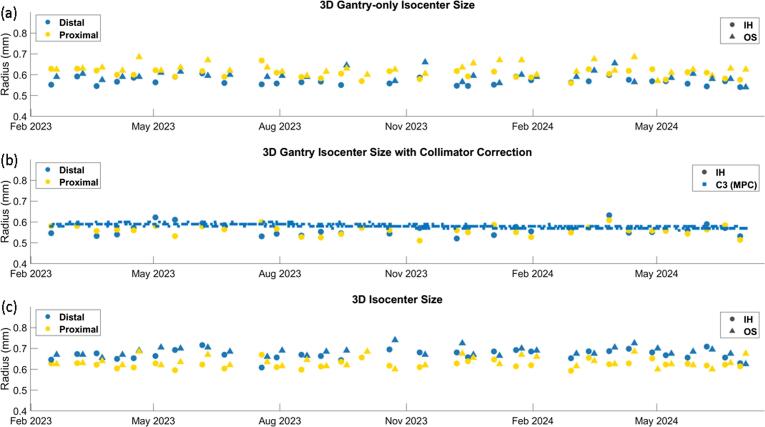
3D gantry isocenter size (a) without or (b) with collimator-walkout correction, and (c) 3D isocenter size, calculated from proximal (yellow) or distal (blue) bank, compared with OS- or C3-reported isocenter size. *IH = in-house Matlab script, C3 = MPC, OS = Pylinac. (For interpretation of the references to colour in this figure legend, the reader is referred to the web version of this article.)

Thresholds for warning and failure offsets were approximately 1.1 mm and 1.3 mm for 2D offsets and 1.0 mm and 1.1 mm for 3D offsets. All three 3D isocenter size metrics are shown in Fig. 3, together with values reported by C3 and OS. C3 uses only distal-bank images and applies collimator-walkout correction; OS does not report a collimator-walkout-corrected isocenter size. Fig. 3 also highlights the longitudinal stability of these metrics, with values remaining highly consistent over time.

The mean collimator walkout radii for the proximal and distal banks were 0.22 mm and 0.19 mm, respectively. The mean distance between the proximal bank walkout center and the combined-bank walkout center was 0.03 mm, whereas this distance increased to 0.07 mm for the distal bank. Although the distal bank exhibited a smaller walkout radius, its walkout center deviated more from the combined-bank axis, indicating poorer axis alignment relative to the proximal bank. This also explains the increase in 3D isocenter size in the distal bank compared to the proximal bank. With collimator-walkout correction applied to the 3D isocenter size, the effect of collimator axis misalignment and walkout difference is removed.

The 2D offset difference between gantry 0° and gantry 180° in the couch longitudinal direction is 0.95 ± 0.09 mm, which is a large component contributing to the overall isocenter size. Unlike C-arm linacs, gantry 0° consistently points couch-out and gantry 180° points couch-in. At gantry 90° and 270°, the beam always points upward due to gravitational sag.

### 3.3 Collimator walkout dependence on gantry angle

**Fig. 4:**
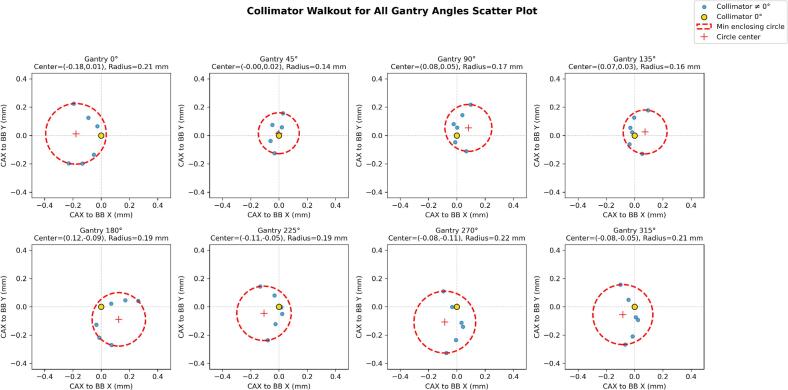
Gantry-specific 2D collimator walkout. At each gantry angle, eight collimator-angle images (blue dots) were acquired and the smallest enclosing circle (red circle) was found. The center and radius of the circle are shown above each panel. (For interpretation of the references to colour in this figure legend, the reader is referred to the web version of this article.)

The 2D collimator walkout at different gantry angles is shown in Fig. 4. The 2D collimator walkout at different gantry angles is similar. P-values for the Mann–Whitney *U* test in all groups were larger than 0.2, which indicates the 2D collimator walkout defined at gantry 0° is not statistically different from that at other gantry angles. The smallest enclosing circle found from the gantry-specific 2D collimator walkout centers is in the supplemental material. There is no significant difference between pre- and post-3D-offset shifts’ image sets. However, the proximal bank showed a slightly larger circle (radius of 0.19 mm) compared to the distal bank (radius of 0.16 mm).

## 4 Discussion

This study observed that the evaluated WL analysis software produced clinically similar results, with the caveat that care must be taken when processing low-contrast images. Institutions should independently characterize their own ring-gantry linacs when performing isocentricity assessments, and the proximal and distal MLC banks should be evaluated separately to fully capture bank-specific isocentricity behavior. The isocentricity values measured for the Halcyon machines in this study are consistent with prior work, which reported 3D offsets of 0.54–0.55 mm [4], [5]. Oare et al., using a conical scintillator system (XRV-124, Logos Systems), similarly observed a 3D isocenter size of 0.70 mm [3].

The C1 outliers reflect its handling of low-contrast images in the presence of a silicon nitride BB. Although silicon nitride minimizes CBCT streaking, it provides less contrast for some edge-detection algorithms [12]; Institution 2 optionally used a tungsten-BB phantom after July 2023, which reduced this variability. More broadly, the threshold-based (C2), histogram-based (OS), and Hough-transform-based (IH) field-edge algorithms differ in their sensitivity to image noise, threshold selection, and beam asymmetry, and these algorithmic differences likely account for the residual sub-millimeter variability observed across software. C1 and C3 use proprietary approaches. Although C1 performed poorly with low-density BBs, its accuracy improved with tungsten BBs, becoming comparable to other tools.

Gantry flex on Halcyon shows a consistent directional pattern, with the pronounced gantry walkout in the couch longitudinal direction most likely attributable to the intrinsic mass imbalance of the ring-gantry design. Gantry 0° consistently points couch-out and gantry 180° points couch-in, opposite to the behavior of C-arm linacs.

Our findings indicate proximal and distal MLC banks exhibit intrinsically different isocentricity behaviors that are driven by geometry: the larger apparent walkout of the proximal bank is consistent with geometric magnification effects from its position closer to the source, whereas the distal bank shows poorer alignment of its walkout axis relative to the combined-bank axis, contributing to a larger uncorrected 3D isocenter size. The angular stability of collimator walkout supports applying a single collimator-walkout correction across gantry angles. Once this correction is applied, bank-specific walkout and setup-uncertainty contributions are largely removed, yielding a collimator-walkout-corrected 3D isocenter size that reflects an intrinsic machine characteristic—consistent with the MPC framework, which explicitly decouples gantry–collimator walkout from phantom misalignment. These observations support performing WL analyses separately for proximal and distal banks to fully capture bank-specific isocentricity performance while maintaining efficient workflows.

Unlike MPC, which uses a drum phantom with multiple BBs to solve for 6-DoF phantom setup [13], our method assumes the CAX is perpendicular to the measurement plane. This explains why the C3-reported isocenter size in Fig. 3 is more stable than IH. Although other studies have incorporated markers at varying distances to account for beam deflection [[14], [15]], we intentionally used a single-BB design for simplicity. The resulting difference remained small (0.1 mm).

Fig. 3 demonstrates the machine isocentricity stability over time, indicating that under normal operating conditions, WL results are highly stable and should not exhibit sudden degradation. Therefore, a failure exceeding warning or tolerance limits is unexpected and likely indicative of a mechanical or system-related issue requiring attention. When WL results exceed the warning or failure thresholds, a scheduled machine service evaluation is warranted, particularly in cases where mechanical components can be physically adjusted by vendor service. In the interim, the clinical impact—especially for small-target treatments—should be carefully assessed. Margins used in clinical planning should be re-evaluated with heightened scrutiny. Additionally, delivery-based patient-specific QA (PSQA) should be performed and rigorously reviewed.

Although proximal–distal differences were statistically significant, they were small (∼0.1 mm) and may have limited clinical impact depending on target size. However, both the imaging-to-radiation isocenter coincidence (3D offset) and the radiation isocenter size should factor into margin selection. These values are intended as institution- and workflow-dependent considerations rather than broadly applicable guidance; clinicians should choose targeting margins based on their own machine characteristics and clinical workflow. For the tested machines, which showed mean imaging-to-radiation isocenter coincidence of 0.8 mm and radiation-isocenter size of 0.7 mm, a minimum targeting margin of 1.5 mm would be appropriate to account for geometric uncertainty. Additional treatment margin should also account for couch-movement uncertainty, image registration uncertainty, intra-fraction motion, surface guidance uncertainty, and contouring uncertainty.

A limitation of this study is that it did not explicitly account for the interplay between the proximal and distal banks when both banks are used simultaneously to define the treatment field. This limitation is particularly relevant for small-field and stereotactic applications, where dosimetric quantities such as output factors, penumbra width, and dose gradients are highly sensitive to submillimeter geometric uncertainties. For fields shaped by both banks, the field dimension in the x direction is defined by the opposing leaf ends of the proximal and distal banks, whereas the y dimension is determined by the leaf edge of a single bank. In the present work, walkout axis alignment and walkout radii were evaluated for each bank independently, allowing isolation of bank-specific geometric behavior; however, these measurements do not directly represent the composite isocentricity or effective field geometry when both banks shape the aperture. As a result, the extent to which single-bank walkout translates into dosimetric deviations remains unclear. Future work will focus on composite WL measurements using both banks simultaneously, as well as dedicated small-field dosimetric studies to quantify the impact of combined-bank geometric behavior on clinically relevant dosimetric endpoints.

This comprehensive dual-bank, multi-institution, multi-software evaluation demonstrates that ring-gantry linacs exhibit stable and reproducible isocentricity across a range of WL-derived metrics. Software-dependent differences were minimal after excluding a single application affected by low-contrast images, and collimator-walkout-corrected 3D isocenter size remained stable—confirming its role as a machine-intrinsic metric independent of MLC bank or phantom setup. The proximal and distal MLC banks showed small but statistically significant differences in walkout behavior, supporting the need for bank-specific WL evaluation. These findings also lay the groundwork for evaluating next-generation systems.

## CRediT authorship contribution statement

**Xiaodong Zhao:** Writing – original draft, Methodology, Investigation, Formal analysis, Data curation, Conceptualization. **Phillip D. Wall:** Writing – review & editing, Investigation. **Justin Mikell:** Writing – review & editing, Methodology, Investigation. **Matthew C. Schmidt:** Writing – review & editing. **Eric Laugeman:** Writing – review & editing. **Cory S. Knill:** Writing – review & editing. **Dennis N. Stanley:** Writing – review & editing. **Richard A. Popple:** Writing – review & editing, Supervision. **Thomas R. Mazur:** Writing – review & editing, Supervision, Methodology, Investigation.

## Declaration of competing interest

The authors declare that they have no known competing financial interests or personal relationships that could have appeared to influence the work reported in this paper.

## Appendix A Supplementary data

Supplementary Data 1 Provides full definitions, equations, and schematic figures (Figs. S1–S9; Table S1) for every Winston–Lutz metric reported in the main text, together with pre- and post-3D-shift warning/failure thresholds (Table S2), pairwise software-to-software 2D-offset comparisons (Tables S3–S4, Fig. S10), proximal- and distal-bank gantry-specific collimator-walkout fit circles (Figs. S11–S12), and an independent simulation-based ground-truth validation of the in-house (IH) and open-source (Pylinac) algorithms.
