# Supplementary material for: Multi-institutional evaluation of isocentricity on a ring-gantry linear accelerator platform with double-stacked MLCs
Source: Phys Imaging Radiat Oncol. 2026 Jun 2;39:101008. doi: 10.1016/j.phro.2026.101008 (PMC13263672; doi:10.1016/j.phro.2026.101008)
Supplement: Supplementary Data 1 — Provides full definitions, equations, and schematic figures (Figs. S1–S9; Table S1) for every Winston–Lutz metric reported in the main text, together with pre- and post-3D-shift warning/failure thresholds (Table S2), pairwise software-to-software 2D-offset comparisons (Tables S3–S4, Fig. S10), proximal- and distal-bank gantry-specific collimator-walkout fit circles (Figs. S11–S12), and an independent simulation-based ground-truth validation of the in-house (IH) and open-source (Pylinac) algorithms. [file mmc1.pdf]

Supplemental Material

1. Details on each listed metric in Table 1 are explained in detail in terms of the calculation procedure, its meaning, and a figure for illustration in Table S1.

Table S1. In-house software parameter definitions, calculation procedures, and schematic illustrations.

Notation: *u, v* = EPID detector axes; *x, y, z* = IEC machine coordinates; *θ, ψ, φ* = gantry, collimator, and table angles, respectively.

Parameter (Availability)	Calculation Procedure	Schematic Figure
**2D Offset**<br>IH, C1 (Inst. 2), C2, OS	**Calculation**<br>For each image, the radiation field centroid in blue (u_f, v_f) and BB centroid in yellow (u_{BB}, v_{BB}) are detected in the EPID projection image. The 2D offset vector is their difference:<br>$\delta 2D = (u_f - u_{BB},\ v_f - v_{BB})$ (S1)<br>where *u* and *v* are the horizontal and vertical axes of the EPID detector. The magnitude $\|\delta 2D\|$ is reported in mm.<br>**Interpretation**<br>Quantifies the planar misalignment between the radiation-field center and the BB position in each portal image.	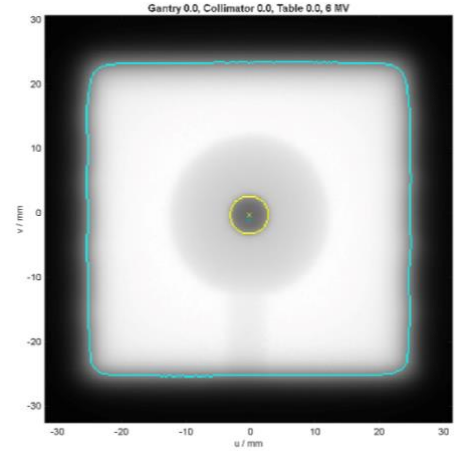<br>***Fig. S1***. *Schematic of 2D offset measurement in the EPID plane, BB centroid (yellow) to the radiation-field centroid (blue).*

Parameter (Availability)	Calculation Procedure	Schematic Figure
**3D Offset**<br>IH, C1 (Inst. 2), C2, OS	**Calculation**<br>Using multiple gantry and collimator angle combinations, the set of 2D offset vectors (δu, δv) are assembled into a linear system. For each beam with gantry angle θ and table angle φ, the projection matrix A is:<br>$A_i = \begin{bmatrix} -cos\varphi & -sin\varphi & 0 \\ -cos\theta\ sin\varphi & cos\theta\ cos\varphi & -sin\theta \end{bmatrix}$ (S2)<br>The 3D BB-displacement vector is obtained by least-squares:<br>$\vec{\delta}_{3D} = (A^TA)^{-1} A^T \cdot \vec{\delta}_{2D}$ (S3)<br>implemented following Low et al. [1]. The magnitude $\|\delta 3D\|$ is reported in mm.<br>**Interpretation**<br>The 3D translation of the BB in the IEC machine coordinate system that minimizes all 2D projection offsets simultaneously. Represents the physical BB displacement from the ideal radiation isocenter.	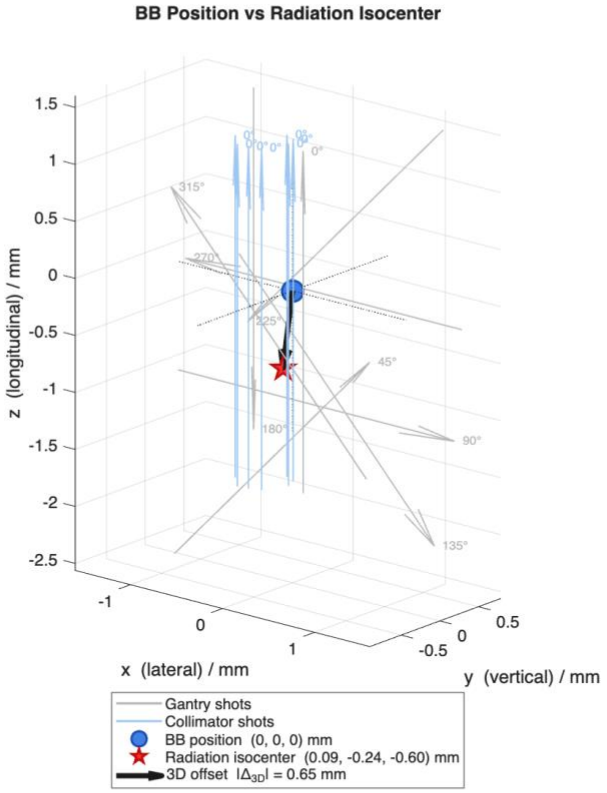<br>***Fig. S2**. 3D offset δ3D (black arrow) from the BB centroid (blue) to the radiation isocenter (red star), estimated by least-squares back-projection of multiple 2D offsets (gray and blue rays).*
**2D Collimator Walkout**<br>IH, OS	**Calculation**<br>With gantry fixed at 0°, images are acquired at multiple collimator angles ψ_i. The 2D offset at each angle relative to the collimator 0° position is computed:<br>$\Delta_i = \delta 2D(\psi_i) - \delta 2D(\psi = 0°)$ (S4)<br>The smallest enclosing circle of the set {Δi} is computed using Welzl's algorithm [2]:<br>$r = min \{ R : \|\Delta i - C\| \leq R, \forall i \}$ (S5)<br>The radius r (mm) is the 2D collimator walkout.<br>**Interpretation**<br>Characterizes the circular path traced by the radiation-field center in the EPID plane as the collimator rotates. A larger r	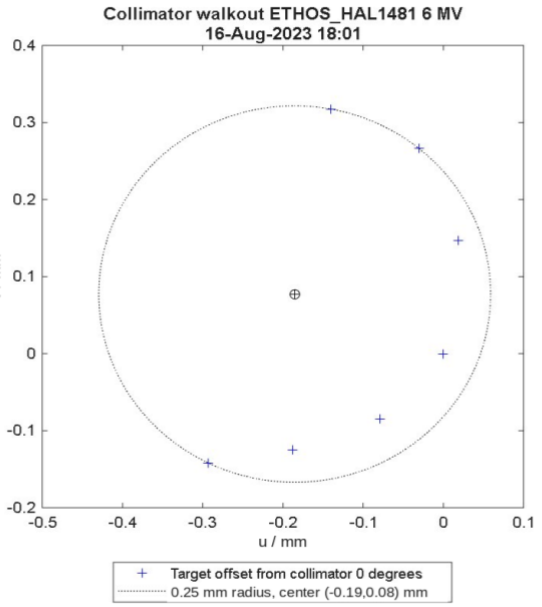<br>***Fig.S3.** Minimum enclosing circle (radius r) of 2D offsets acquired at each collimator angle with*

Parameter (Availability)	Calculation Procedure	Schematic Figure
	indicates greater collimator mechanical wobble.	*gantry at 0°. Each point represents one collimator angle.*
**2D Gantry Walkout**<br>IH only	**Calculation**<br>With collimator fixed at 0°, images are acquired at multiple gantry angles θ_i. The minimum enclosing circle fit with Welzl's algorithm [2] is found:<br>$r = min \{ R : \lVert \Delta i - C \rVert \leq R, \forall i \}$ (S6)<br>**Interpretation**<br>Characterizes the circular spread of radiation field centers in the EPID plane as the gantry rotates (collimator held at 0°). Analogous to 2D collimator walkout but attributed specifically to gantry rotation mechanics.	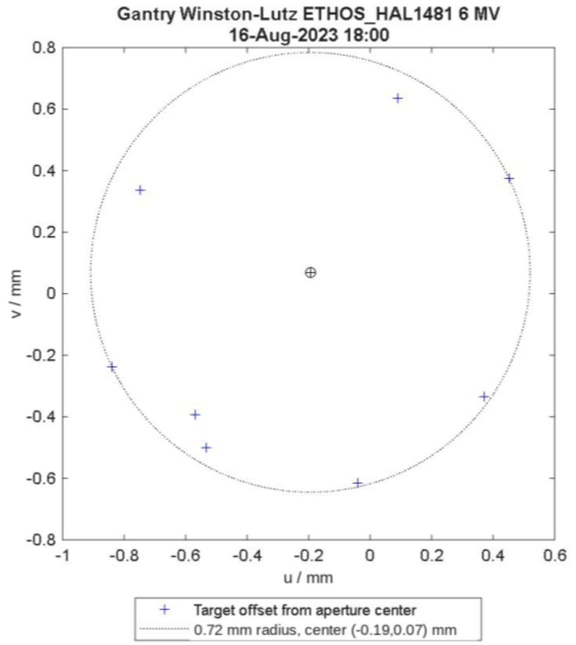<br>***Fig.S4.*** *Minimum enclosing circle (radius r) of 2D offsets acquired at each gantry angle with collimator at 0°. Each point represents one gantry angle.*

<table>
<tr><th>Parameter (Availability)</th><th>Calculation Procedure</th><th>Schematic Figure</th></tr>
<tr>
<td>3D Gantry-only Isocenter Size
IH, OS</td>
<td>

Calculation

Using only gantry-rotation images (collimator = 0°), the central axis (CAX) of each beam is reconstructed as a **3D ray** with the BB placed at the origin. For a gantry angle θi and measured 2D offset (ui, vi), the CAX ray is parameterized by a point pi and direction di:

$$p_i = \left(0,\ v_i,\ -\frac{u_i}{\sin\theta_i}\right) \quad (S7)$$

$$d_i = (\sin\theta_i,\ 0,\ \cos\theta_i)(S8)$$

The isocenter size R is defined as the radius of the smallest sphere that is tangent to (or encloses) all back-projected CAX rays. Equivalently, its center C minimizes the maximum perpendicular distance from C to any ray:

$$R = \min_C \max_i \frac{\|(C - p_i) \times d_i\|}{\|d_i\|} \quad (S9)$$

Interpretation

The radius of the smallest 3D sphere through which all gantry CAX rays pass. Reflects the 3D volumetric uncertainty of the radiation isocenter due solely to gantry-rotation mechanics.

</td>
<td>

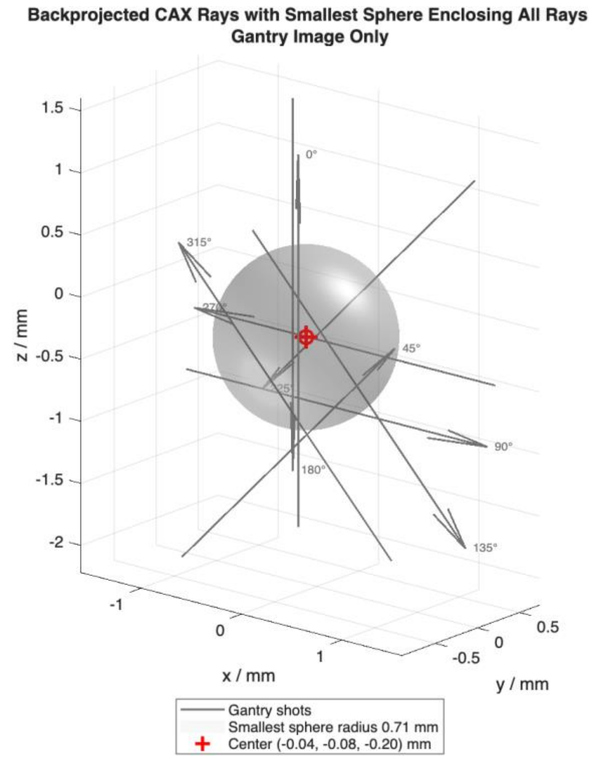

Fig. S5. *Schematic 3D gantry-only isocenter size: gray lines are back-projected CAX rays from gantry-only shots; the gray sphere (radius R, center +) is the smallest sphere tangent to or encompasses all rays.*

</td>
</tr>
</table>

Parameter (Availability)	Calculation Procedure	Schematic Figure
**3D Gantry Isocenter Size with Collimator Walkout Correction**<br>IH, C3	**Calculation**<br>Calculated identically to the 3D gantry-only isocenter size (Eqs. S7–S9), except that each gantry image's 2D offset is first corrected for the collimator walkout contribution at its actual collimator angle ψi:<br>$$\delta_{2D,corr}(\theta_i) = \delta_{2D}(\theta_i) - \Delta_{coll}(\psi_i) \text{(S10)}$$<br>where Δcoll(ψi) is the displacement vector (blue arrow in Fig. S6) from the collimator-0° position to the collimator-walkout-circle center (derived from the 2D collimator walkout analysis). The corrected offsets are then back-projected (Eqs. S7, S8) and the smallest tangent sphere is computed (Eq. S9):<br>$$R_{corr} = \min_{C} \max_{i} \frac{\|(C - p_{i,corr}) \times d_i\|}{\|d_i\|}$$ (S11)<br>This parameter is directly comparable to the Machine Performance Check (MPC) **isocenter size** reported by the Varian system. In MPC, the input imaging set consists of beam-center-check images (5 collimator-rotation images) plus IsoCal images (16 gantry images at 8 gantry angles, each with an MLC comb pattern), using the distal MLC bank only.<br><br>**Interpretation**<br>Isolates the gantry-rotation contribution to 3D isocenter uncertainty by removing the collimator-walkout component. Provides the estimate most directly comparable to the MPC isocenter size.	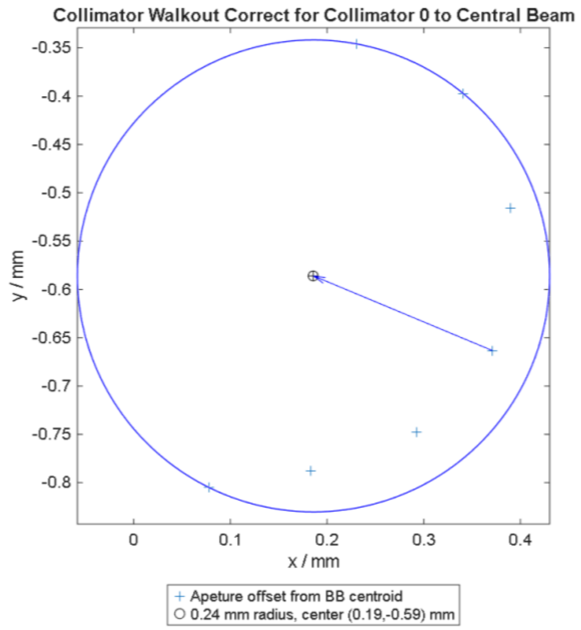<br><br>***Fig. S6.*** *A collimator walkout compensation vector (blue arrow) was decided by the collimator images alone at gantry 0° from the collimator-0° position to the collimator-walkout-circle center.*<br>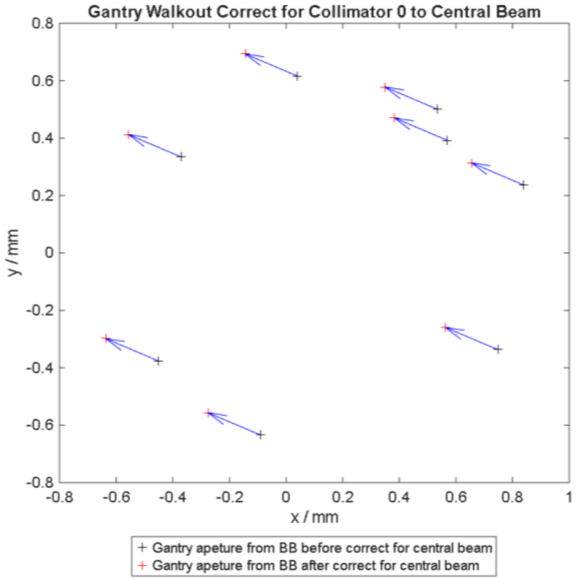<br><br>***Fig. S7*** *The collimator walkout compensation vector was applied at each gantry angle such that*

Parameter (Availability)	Calculation Procedure	Schematic Figure
		the collimator walk effect was removed.<br>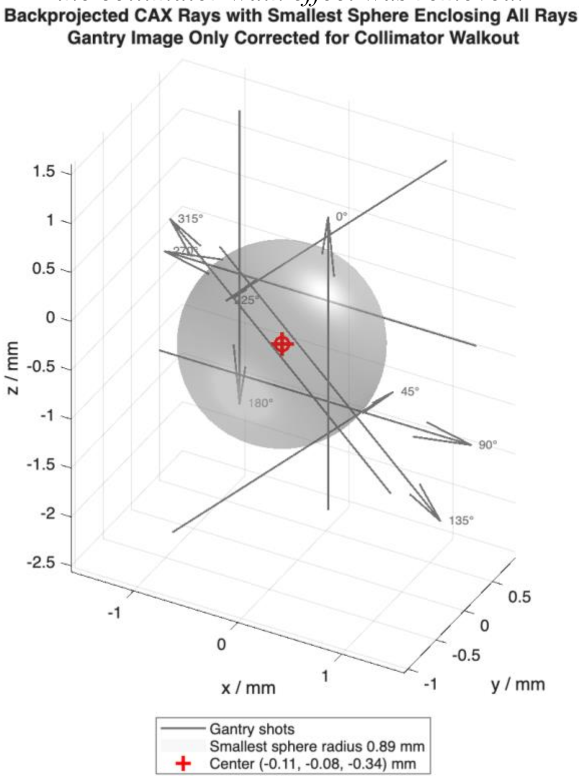<br><br>***Fig. S8.*** *Schematic 3D gantry-only isocenter size with collimator-walkout correction. CAX rays are corrected for collimator walkout and then back-projected.*

Parameter (Availability)	Calculation Procedure	Schematic Figure
**3D Isocenter Size**<br>IH, OS	**Calculation**<br>All portal images—both collimator-rotation and gantry-rotation shots—are included. Each CAX ray is reconstructed as a 3D line (pi, di) using Eqs. S7–S8. The smallest tangent sphere enclosing all N rays is then computed:<br>$R_{tot} = \min_{C} \max_{i \in \{gantry \cup coll\}} \frac{\lVert (C - p_i) \times d_i \rVert}{\lVert d_i \rVert}$ (S13)<br>**Interpretation**<br>This isocenter-size metric includes all delivered shots. It captures the combined effect of all mechanical degrees of freedom (gantry rotation and collimator rotation) on the 3D stability of the radiation isocenter.	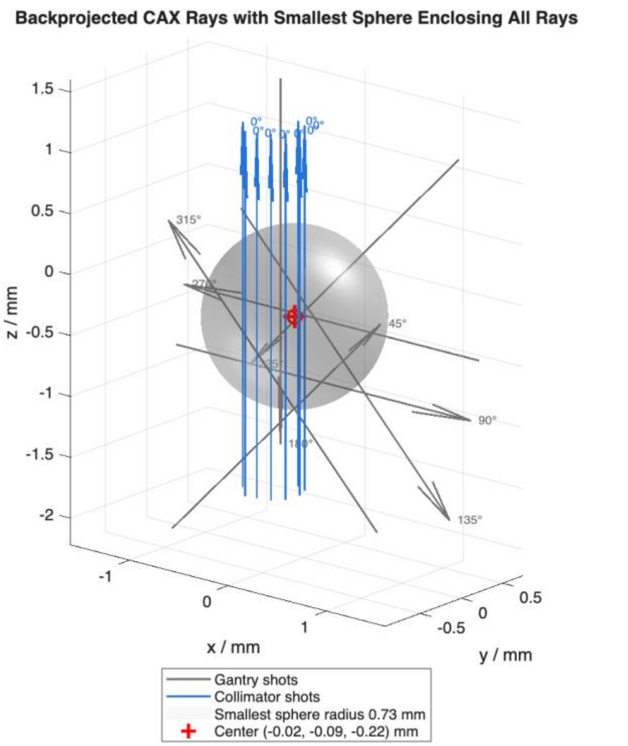<br>***Fig. S9.*** *3D isocenter size calculated from both back-projected CAX rays in collimator shots in blue and gantry shots in gray.*

2. Warning and failing threshold of 2D offset

The warning and failing threshold of 2D offset magnitude based on 1.96σ and 3σ from mean value is 1.1mm and 1.4mm among IH, C2 and OH from institution 1, considering all 2D data acquired. In institution 2, the warning and failing threshold is 1.1mm and 1.3mm among IH, C2 and OH, and 1.1mm and 1.4mm for C1, only considering the high-density BB images. If separating the pre-3D shift and post-3D shift results, the warning and failing levels are tabulated in Table 1.

Table S2. Warning and Failing levels of 2D offset magnitude in mm.

		Pre-3D shift							Post-3D shift						
		IH		OS		C1	C2		IH		OS		C1	C2	
		Inst1	Inst2	Inst1	Inst2	Inst2	Inst1	Inst2	Inst1	Inst2	Inst1	Inst2	Inst2	Inst1	Inst2
Proximal bank	Warning	1.4	1.1	1.4	1	0.9	1.4	1	0.8	0.9	0.8	1	1.3	0.8	1
	Failing	1.7	1.3	1.7	1.2	1	1.7	1.2	1	1.2	1	1.2	1.7	1	1.2
Distal bank	Warning	1.1	1.2	1.1	1.1	0.9	1.1	1.2	0.8	1	0.9	1.1	1	0.9	1
	Failing	1.3	1.4	1.4	1.4	1	1.4	1.4	1.1	1.3	1.1	1.3	1.3	1.1	1.3

*Inst1 = Institution 1, Inst2 = Institution 2, IH=in-house Matlab script, C1=Total QA, C2=DoseLab, OS=Pylinac

3. 2D offset

Table S3. 2D offset differences between applications

2D Offset Differences (Mean [Min, Max], mm)				
**Institution**	**Comparison**	**x (mm)**	**y (mm)**	**Magnitude (mm)**
**1**	OS vs IH	0.03 [0.00, 0.13]	0.04 [0.00, 0.21]	0.04 [0.00, 0.21]
	C2 vs IH	0.03 [0.00, 0.13]	0.04 [0.00, 0.21]	0.03 [0.00, 0.21]
**2**	OS vs IH	0.02 [0.00, 0.11]	0.03 [0.00, 0.18]	0.03 [0.00, 0.15]
	C1 vs IH	0.08 [0.00, 0.74]	0.08 [0.00, 0.99]	0.09 [0.00, 0.88]
	C2 vs IH	0.02 [0.00, 0.10]	0.03 [0.00, 0.13]	0.03 [0.00, 0.12]

C1 exhibited noticeably greater uncertainty than all other applications.

Table S4. 2D Offset Magnitude Pre- and Post-3D-shift

2D Offset Magnitude (Mean ± SD, mm) Pre- and Post-3D-Shift			
**Institution**	**Bank**	**Pre-3D-Shift (mm)**	**Post-3D-Shift (mm)**
**1**	Distal	0.67 ± 0.23	0.45 ± 0.21
	Proximal	0.75 ± 0.31	0.45 ± 0.18
**2**	Distal	0.70 ± 0.23	0.54 ± 0.26
	Proximal	0.67 ± 0.19	0.53 ± 0.21

In both institutions and in both banks, the 3D shift consistently reduced the 2D offset magnitude.

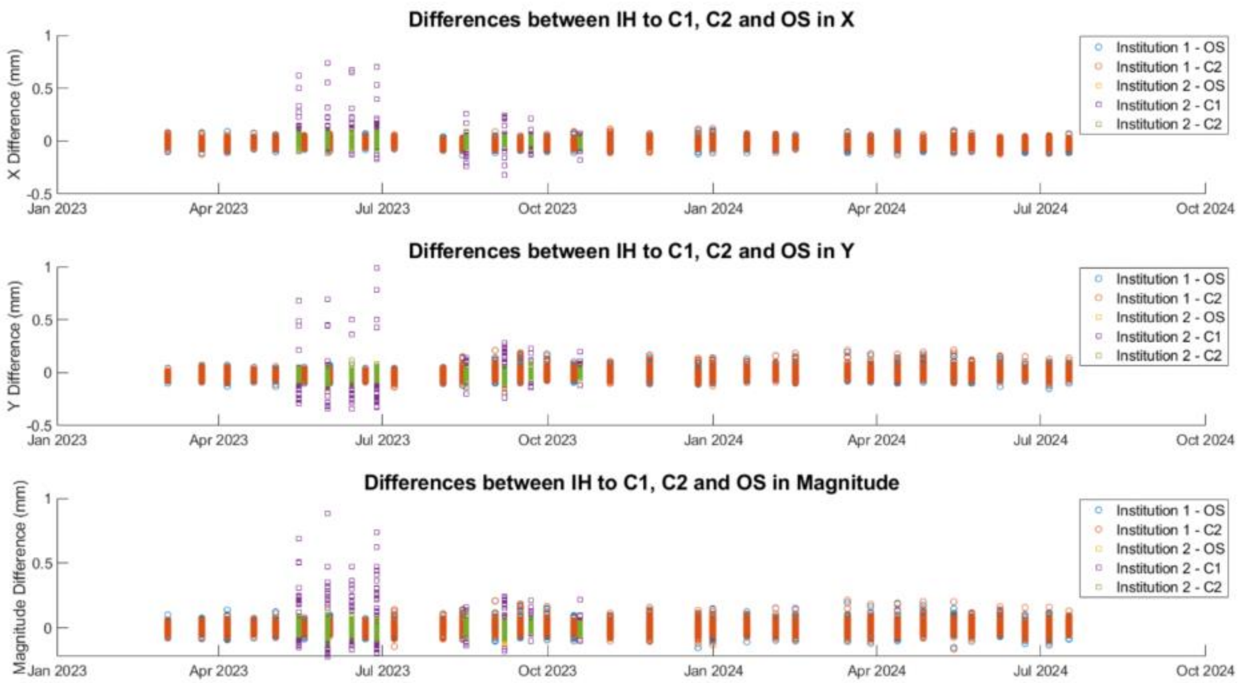

Figure S10. 2D offset differences between C1, C2, and OS compared to IH. The largest difference is shown in C1, which is due to the incorrect detection of BB due to low density BB's image contrast. Low-density BB was used before July 2023 and high-density BB afterwards in Institution 2. *IH=in-house Matlab script, C1=Total QA, C2=DoseLab, OS=Pylinac

4. 2D Collimator Walkout and 2D Gantry Walkout

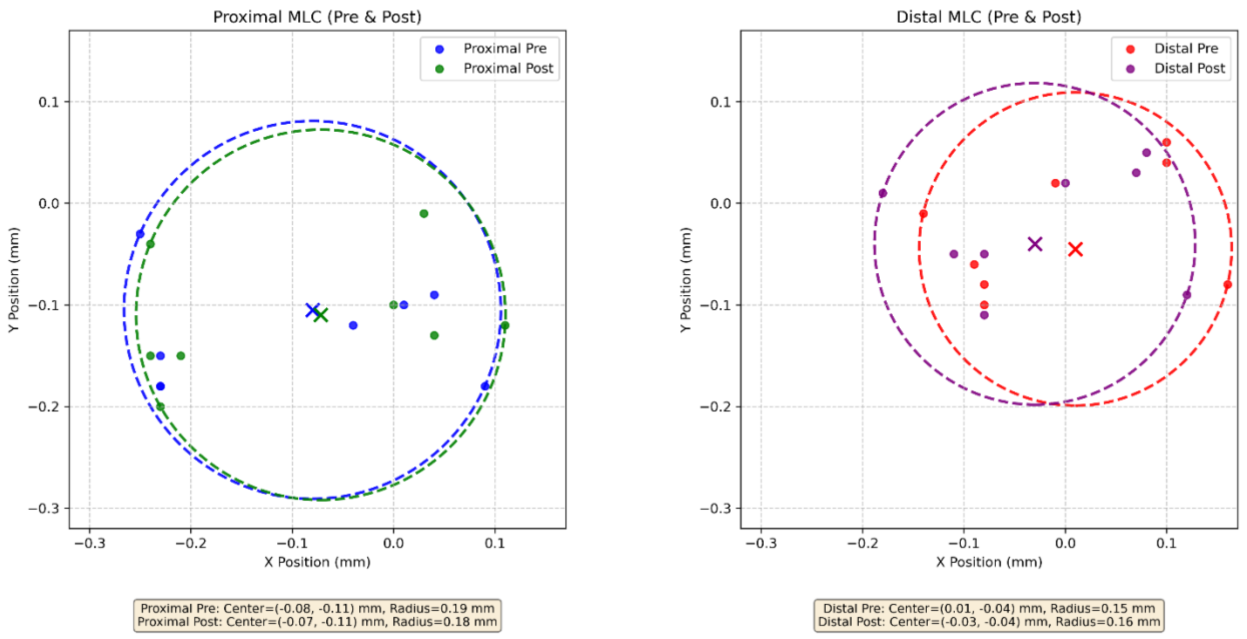

Figure S11. The smallest enclosing circle found from the gantry-specific walkout centers in proximal pre-3D-offset, proximal post-3D-offset, distal pre-3D-offset, and distal post-3D-offset image sets. Each dot is from the centers found in the gantry-specific 2D collimator walkout. X is the center of the fit circle.

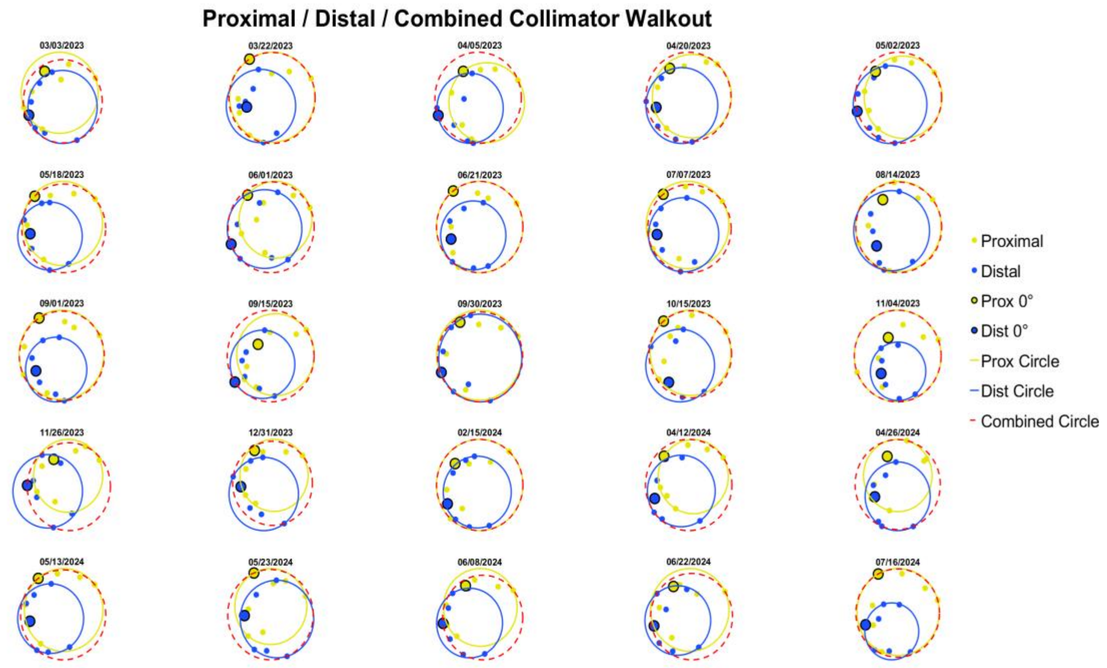

Figure S12. Proximal and distal bank collimator walkout and combined walkout. Proximal walkout showed larger radius but more centered and distal walkout showed smaller radius but less centered.

5. Using simulated images to test the ground truth of the IH and OS algorithms

An independent ground truth validation using simulated EPID images with known, prescribed geometric offsets was described below in four steps.

First, we tested the repeatability of the algorithm to noise in the images being processed. Without moving the BB, MLCs, gantry, or collimator, we delivered 15 images consequently. For locating the BB center and field center, the analysis algorithms demonstrated variability of min to max over these 15 realizations with a standard deviation of 0.01mm in BB center and field center.

Second, we simulated an ideal Winston-Lutz scenario in which the BB is perfectly centered at the radiation isocenter, with no collimator walkout, no gantry walkout, and an isocenter size of 0 mm (collapsed to a point). Both tools were applied to these images. The IH tool detected a radiation isocenter position of (0.03, 0, 0) mm, with gantry-only isocenter size, gantry-only isocenter size with collimator correction, and full 3D isocenter size all equal to 0.04 mm. Additionally, the IH tool measured collimator and gantry walkout as 0.00 mm. The OS tool returned 0.00 mm for all metrics. These results confirm that both tools return results within 0.05 mm of expected values under ideal conditions.

Third, to validate the 2D walkout function specifically, we simulated a set of collimator images in which the radiation field center was prescribed to trace a circle of radius 0.5 mm, with the BB fixed at the image center. Images were generated at collimator angles from 90° to 270° in 30° increments. When these images were analyzed using only the 2D walkout function in isolation, the IH tool reported a collimator walkout radius of 0.50 mm and the OS tool reported 0.52 mm, both within 0.02 mm of the prescribed ground truth of 0.50 mm.

Fourth, to validate the 3D sphere reconstruction function specifically, we simulated a set of gantry images in which the radiation field center was prescribed to trace a sphere of radius 0.5 mm as the gantry rotated, equivalent to a gantry up-down flex of 1.0 mm in the gun-target direction. When these images were analyzed using only the 3D reconstruction function in isolation, the IH tool reported a 3D isocenter radius of 0.53 mm and the OS tool reported 0.46 mm, corresponding to errors of +0.03 mm and −0.04 mm respectively relative to the ground truth.

Across all three simulated validation scenarios, both tools demonstrated sub-millimeter agreement with the prescribed ground truth, with maximum deviations of 0.04 mm under ideal conditions and 0.04 mm for the 3D reconstruction. These results provide independent, simulation-based corroboration of the accuracy of both the IH and OS implementations.

References

[1] Lutz W, Winston KR, Maleki N. A system for stereotactic radiosurgery with a linear accelerator. Int J Radiat Oncol Biol Phys 1988;14:373–81. https://doi.org/10.1016/0360-3016(88)90446-4

[2] Welzl E. Smallest enclosing disks (balls and ellipsoids). In: Maurer H, editor. New Results and New Trends in Computer Science. Lecture Notes in Computer Science, vol. 555. Berlin: Springer; 1991. p. 359–70. https://doi.org/10.1007/BFb0038202

[3] Winkler P, Bergmann H, Stuecklschweiger G, Guss H. Introducing a system for automated control of rotation axes, collimator and laser adjustment for a medical linear accelerator. Phys Med Biol 2003;48:1123–32. https://doi.org/10.1088/0031-9155/48/9/303
